# Quantification of aortic pulse wave velocity in preterm infants using 4D phase contrast MRI

**DOI:** 10.1186/1532-429X-15-S1-M7

**Published:** 2013-01-30

**Authors:** Kathryn Broadhouse, Anthony N Price, Giuliana Durighel, Anna Finnemore, David J Cox, AD Edwards, Joseph V Hajnal, Alan Groves

**Affiliations:** 1 Imaging Sciences Department, MRC Clinical Sciences Centre, Imperial College, Hammersmith Hospital, London, UK; 2 The Centre for the Developing Brain, Imaging Sciences & Biomedical Engineering Division, St Thomas' Hospital, King's College, London, UK

## Background

4D phase contrast (PC) MRI sequences providing full coverage of the aortic arch were acquired in neonates. Aortic pulse wave velocity (PWV) was then calculated from flow measurements taken at 5 to 8 locations along the arch and the aortic length between each location. Neonatal PWV values were compared with previously published adult values.

Mechanical compliance within the healthy and diseased aorta has been well documented in adults and paediatrics [1,2]. Metafratzi et al [1] reported a PWV range from 4 to 10 ms-1 in the healthy adult aorta, whilst Vulliemoz et al found a mean PWV of 4.4 ms-1 [3]. PWV is an inverse measure of vessel compliance and marker for vessel stiffness. A significant increase in PWV has been found in paediatric subjects born prematurely at low birthweight when studied at ~8yrs and may explain the increase in cardiac disease in this population[2]. However PWV models do not extend back to preterm infants. Recent studies in adults have used PC MRI flow data to determine PWV [4].

## Objective

Assess feasibility of measuring aortic PWV in preterm and term infants using 4D PC MRI, in order to establish a normative range in this population and facilitate comparison with adult values.

## Methods

**Figure 1:**
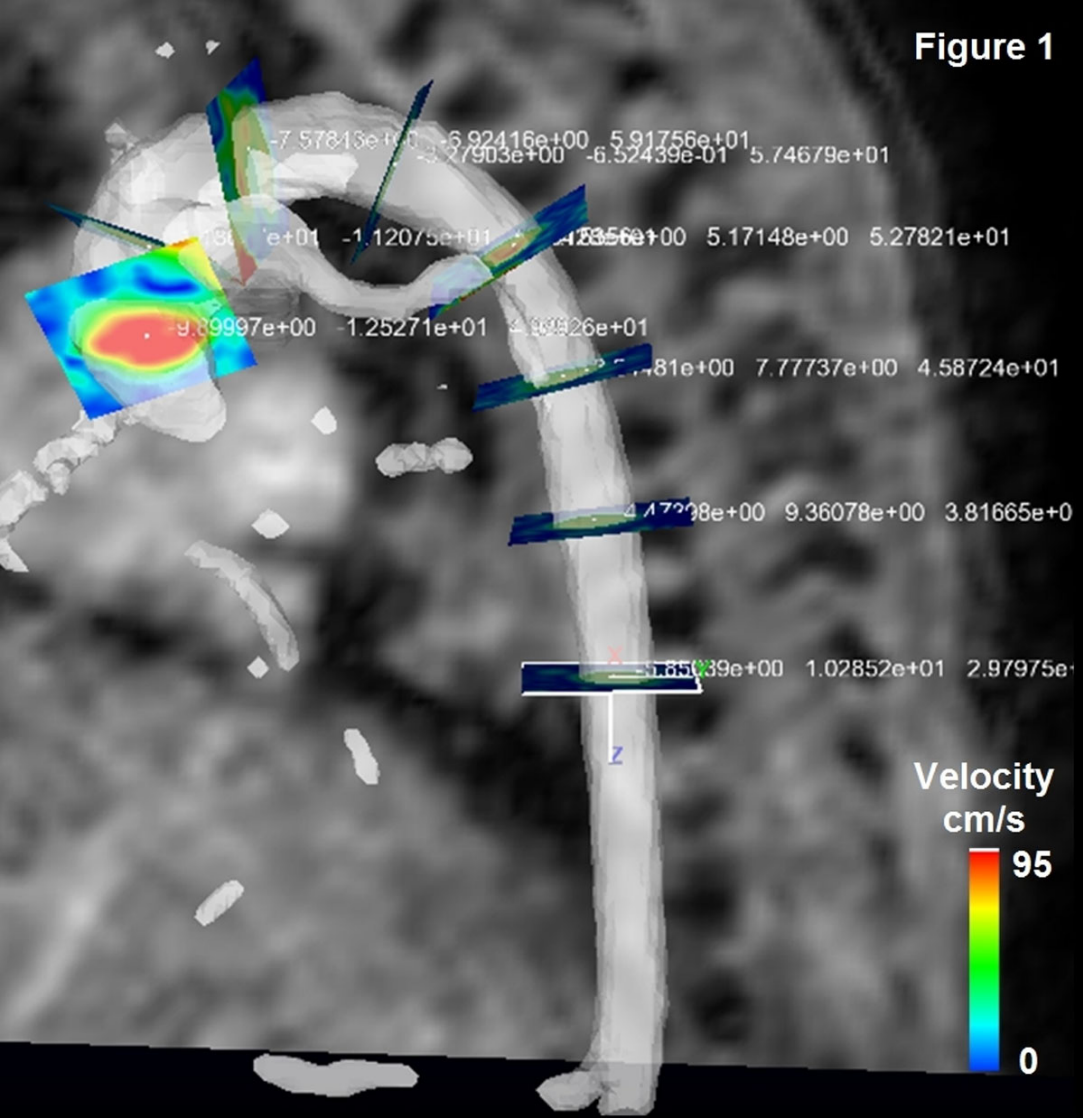
Location of 8 clip planes are shown with iso-volume rendering of aorta in an 875g infant.

Scans were performed on a Philips 3T MR scanner and pediatric or extremities coil. Infants were scanned with ear protection, routine monitoring and without sedation/anesthesia. Between 5 and 8 planes were positioned along the aorta from aortic valve to descending aorta at the level of the diaphragm orthogonal to the lumen using a commercially available software (EnSight; CEI, Apex, NC, USA) (Figure 1) and then exported. Flow was then quantified at these locations in (using flow_tool, MathWorks Matlab [5]). Time delay was calculated by performing a least squares fit (using MathWorks Matlab) between 1st and scaled upslope regions of the consecutive planes (defined as time between 15% and 90% of upstroke flow). The aortic length was determined from interpolation between the centre of the lumen's spatial coordinates of each plane (using MathWorks Matlab). Time delay was plotted against aortic distance between the 1st and consecutive planes. PWV (defined as aortic length/time delay) was then obtained from the gradient of the slope.

**Figure 2:**
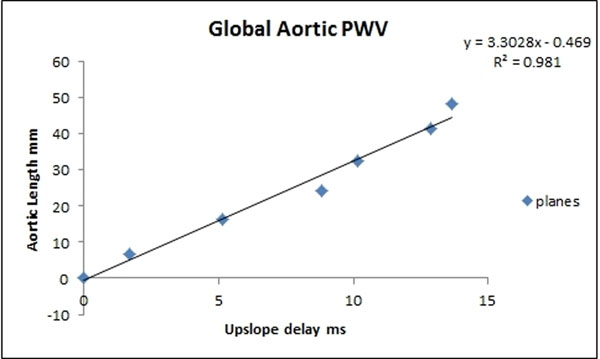
Time delay plotted against aortic distance between the 1st and consecutive planes. PWV (defined as aortic length/time delay) obtained from the gradient of the slope.

## Results

9 infants median (range) corrected GA 32(30+4-36+5) weeks and weight at scan 1330(875-2070) grams were scanned. Mean (range) PWV was found to be 3.2(2.3-5.1)ms-1.

## Conclusions

In this initial study PWV velocity in neonates was found to be at the lower end of reported adult ranges.

## Funding

MRC studentship funding
